# Trade-offs in the genetic control of functional and nutritional quality traits in UK winter wheat

**DOI:** 10.1038/s41437-022-00503-7

**Published:** 2022-04-07

**Authors:** Nick S. Fradgley, Keith Gardner, Matt Kerton, Stéphanie M. Swarbreck, Alison R. Bentley

**Affiliations:** 1 NIAB, 93 Lawrence Weaver Road, Cambridge, CB3 0LE UK; 2 Department of Plant Sciences, University of Cambridge, Downing Street, Cambridge, CB2 3EA UK; 3 DSV UK, Wardington, Banbury, OX17 1FE UK; 4 Present Address: International Maize and Wheat Improvement Center (CIMMYT), El Batán, C.P.56237 Mexico

**Keywords:** Plant breeding, Quantitative trait, Genetic variation

## Abstract

A complex network of trade-offs exists between wheat quality and nutritional traits. We investigated the correlated relationships among several milling and baking traits as well as mineral density in refined white and whole grain flour. Our aim was to determine their pleiotropic genetic control in a multi-parent population over two trial years with direct application to practical breeding. Co-location of major quantitative trait loci (QTL) and principal component based multi-trait QTL mapping increased the power to detect QTL and revealed pleiotropic effects explaining many complementary and antagonistic trait relationships. High molecular weight glutenin subunit genes explained much of the heritable variation in important dough rheology traits, although additional QTL were detected. Several QTL, including one linked to the *TaGW2* gene, controlled grain size and increased flour extraction rate. The semi-dwarf *Rht-D1b* allele had a positive effect on Hagberg falling number, but reduced grain size, specific weight, grain protein content and flour water absorption. Mineral nutrient concentrations were lower in *Rht-D1b* lines for many elements, in wholemeal and white flour, but potassium concentration was higher in *Rht-D1b* lines. The presence of awns increased calcium content without decreasing extraction rate, despite the negative correlation between these traits. QTL were also found that affect the relative concentrations of key mineral nutrients compared to phosphorus which may help increase bioavailability without associated anti-nutritional effects of phytic acid. Taken together these results demonstrate the potential for marker-based selection to optimise trait trade-offs and enhance wheat nutritional value by considering pleiotropic genetic effects across multiple traits.

## Introduction

Wheat is a crop of major global importance (Shiferaw et al. 2013), and contributes to diets in the developed world where food insecurity is rare (Lockyer and Spiro 2020). However, low intake of whole grains is the leading dietary risk factor for deaths in most developed countries (Afshin et al. 2019), and mineral nutritional density has been subject to a dilution effect as yields have increased (Davis 2009; Murphy et al. 2008; Shewry et al. 2016). At the same time as enhancing wheat production to meet the food demands of a rapidly growing global population (Godfray et al. 2010), there is therefore a need to develop high quality wheat varieties for human consumption with increased nutritional value.

Identification and characterisation of genetic regions that are consistently associated with improved functional or nutritional quality would enable improved selection for favourable allele and trait combinations via marker-assisted selection. Significant advances have been made in identifying the genetic control of major quality attributes including high molecular weight (HMW) glutenin and gliadin sub-units on the homoeologous chromosomes 1A, 1B and 1D (Payne and Lawrence 1983). In addition, the differentiation between hard and soft endosperm texture was found to be a result of a genetic effect of puroindoline proteins controlled by two tightly linked genes on chromosome 5D (Morris 2002). These large genetic effects have been readily utilised in breeding programmes. Numerous quantitative trait loci (QTL) mapping studies have detected novel marker trait associations for wheat functional quality (Kristensen et al. 2018; Kuchel et al. 2006; Perretant et al. 2000; Tadesse et al. 2015) and micronutrient content (Guttieri et al. 2015a, b; Liu et al. 2019). However, these typically only explain a small proportion of the variance of the measured trait, are of small effect size, often not stable across environments and their effects not validated in independent genetic backgrounds. Hence, their use in direct breeding programmes has been limited. Looking beyond the well-known major effects, considering genetic effects across a wide range of important traits, and the trade-offs among them, may be a more effective approach for applied wheat breeding.

Many crop traits are known to be genetically correlated so that changes in one trait often result in a correlated response in others due to genetic linkage or pleiotropy (Chen and Lübberstedt 2010). These relationships can either be complementary, where easier to measure or higher heritability traits can be used to indirectly manipulate another desired trait, or antagonistic, where manipulating one trait has a negative effect on another. Here we investigate complementary and antagonistic trait relationships for a wide range of milling and baking quality and mineral density traits and determine their genetic control using an eight-founder Multi-Parent Advanced Generation Inter Cross (MAGIC) population that is representative of elite wheat diversity in the UK (Mackay et al. 2014; Gardner et al. 2016). Multi-parent populations capture greater genetic and phenotypic diversity than bi-parental populations, specifically enabling investigation of the interplay between traits (Scott et al. 2020). A high degree of recombination is achieved without residual population structure (Huang et al. 2015), allowing accurate QTL resolution (Scott et al. 2020; 2021), and consequently better distinction between the effects of pleiotropy and genetic linkage in multi-trait analysis. As we were interested in application of findings in a breeding context, we focused on detection in an unbalanced experimental design assessed at a single location in each of two years. This closely reflects an applied breeding context and has been previously shown to be effective for understanding the genetic basis of wheat quality (Zhang‐Biehn et al. 2021). Based on these experiments we used a data analysis approach based on principal component analysis of multi-trait and multi-environment phenotypes as applied by Zhang et al. (2012) as well as Xei and Sparkes (2021). We detected novel loci in addition to previously characterised genes and characterise their pleiotropic effects to assess their utility for application in marker-assisted wheat breeding.

## Materials and methods

### Plant material and trials

Experiments were conducted using 231 lines from the NIAB Elite MAGIC (MEL) population created through the inter-crossing of eight UK founder varieties (Alchemy, Brompton, Claire, Hereward, Rialto, Robigus, Soissons and Xi-19; Mackay et al. 2014). Double dwarf lines carrying both the *Rht-B1b* and *Rht-D1b* reduced height alleles were already removed during creation of the population (Mackay et al. 2014). For this experiment, we used a selected subset of the population with hard endosperm texture based on genetic markers for the Puroindoline-B (*Pinb*) gene (Giroux and Morris 1998), and absence of the 1BL/1RS translocation from rye (*Secale cereale*), known to have negative effects on quality traits (Peña et al. 1990). We used available genotype data (Mackay et al. 2014) generated using the Illumina 90 K wheat SNP array (Wang et al. 2014), and the genetic map described by Gardner et al. (2016). KASP (Kompetitive allele specific PCR) genotyping (He et al. 2014) for the homoeologous high molecular weight (HMW) glutenin loci (*Glu-1B* and *Glu-1D*), as described by Payne and Lawrence (1983), was also applied across the 231 lines. Field trials were conducted at the Deutsche Saatveredelung (DSV) UK breeding station over two trial seasons, reflecting standard breeding assessment capacity and timeframes. Details of field trials and design are given in Supplementary Text 1.

### End-use quality and mineral nutrient composition trait assessment

A wide range of end-use quality traits and micronutrient concentrations were evaluated in both trial years using methods approved by the American Association of Cereal Chemists (AACC 2000), and are summarised in Table 1, and detailed in Supplementary Text 2.

**Table 1:** A total of 38 traits were measured across 231 MEL lines assessed in field trials over two years using American Association of Cereal Chemists (AACC) approved methods.

Trait	Abbreviation	AACC method	Heritability			Correlation between years (r)
			Year 1	Year 2	Meta	
Grain protein content (%)	GPC	39–25.01	0.81	0.86	0.83	0.63
Specific weight (kg hl^−1^)	SPW	55–10.01	0.89	0.93	0.90	0.82
Grain hardness (SKCS hardness)	SKCS	55–31.01	0.87	0.91	0.81	0.70
Extraction rate (%)	ER	–	0.64	0.76	0.74	0.61
Flour whiteness (Tristimulus L*)	L*	14–22.01	0.79	0.35	0.73	0.40
Flour yellowness (Tristimulus b*)	b*	14–22.01	0.91	0.89	0.86	0.85
Overall flour colour (Tristimulus L*-b*)	L*-b*	14–22.01	0.89	0.61	0.84	0.73
Hagberg Falling Number (s)	HFN	56–81.03	0.54	0.58	0.42	0.28
MARVIN grain area (mm^2^)	GA	–	0.92	0.82	0.89	0.80
MARVIN grain length (mm)	GL	–	0.95	0.88	0.95	0.89
MARVIN grain width (mm)	GW	–	0.91	0.84	0.87	0.78
MARVIN thousand grain weight (g)	TGW	–	0.92	0.88	0.87	0.78
DoughLab Bandwidth at Peak	BWAP	54–70.01	0.53	0.86	0.70	0.49
DoughLab Development time (s)	DT	54–70.01	0.24	0.83	0.48	0.32
DoughLab mixing tolerance index (mNm)	MTI	54–70.01	0.83	0.91	0.77	0.63
DoughLab Peak Energy	PE	54–70.01	0.27	0.81	0.45	0.30
DoughLab Softening	SO	54–70.01	0.80	0.87	0.75	0.61
DoughLab Stability	ST	54–70.01	0.90	0.85	0.72	0.58
DoughLab Water absorption (%)	WA	54–70.01	0.79	0.88	0.77	0.62
SDS Sedimentation (ml)	SDS	54–61.01	0.84	0.64	0.74	0.62
Calcium in white flour (mg kg^−1^)	Ca White		0.62	0.69	0.79	0.63
Calcium in whole meal flour (mg kg^−1^)	Ca Whole		0.75	0.80	0.77	0.58
Iron in white flour (mg kg^−1^)	Fe White		0.48	0.16	0.07	−0.06
Iron in whole meal flour (mg kg^−1^)	Fe Whole		0.27	0.66	0.51	0.31
Potassium in white flour (mg kg^−1^)	K White		0.51	0.54	0.74	0.61
Potassium in whole meal flour (mg kg^−1^)	K Whole		0.49	0.30	0.41	0.31
Magnesium in white flour (mg kg^−1^)	Mg White		0.22	0.36	0.59	0.38
Magnesium in whole meal flour (mg kg^−1^)	Mg Whole		0.65	0.33	0.54	0.40
Manganese in white flour (mg kg^−1^)	Mn White		0.24	0.32	0.54	0.38
Manganese in whole meal flour (mg kg^−1^)	Mn Whole		0.77	0.50	0.60	0.37
Phosphorus in white flour (mg kg^−1^)	P White		0.05	0.42	0.37	0.10
Phosphorus in whole meal flour (mg kg^−1^)	P Whole		0.40	0.31	0.42	0.24
Sulphur in white flour (mg kg^−1^)	S White		0.06	0.55	0.50	0.23
Sulphur in whole meal flour (mg kg^−1^)	S Whole		0.00	0.41	0.41	0.26
Selenium in white flour (mg kg^−1^)	Se White		0.44	0.18	0.07	0.03
Selenium in whole meal flour (mg kg^−1^)	Se Whole		0.44	0.58	0.44	0.01
Zinc in white flour (mg kg^−1^)	Zn White		0.43	0.18	0.19	0.10
Zinc in whole meal flour (mg kg^−1^)	Zn Whole		0.52	0.64	0.49	0.24

Values of broad sense heritability are given for genotype best linear unbiased predictions (BLUPs) calculated within each year as well as across both years in a meta-analysis. The correlation coefficient was also calculated between BLUPs within each year.

### Statistical analysis

Best Linear Unbiased Predictions (BLUPs) were calculated for each trial year as well as in a meta-analysis across years to generate adjusted means for each trait per line. Temporal effects among technical replications across analysis batches and days were found to be negligible so multiple measurements of each plot were averaged and per plot data analysed using Restricted Maximum Likelihood (ReML) mixed effects models with auto regressive spatial models in Genstat, 18th edition (Payne, 2009; VSN International 2019). For each trait, spatial autoregression models were run with ‘line’ as a random effect and all combinations of levels of the sub-blocking structure as a random effect. The best fitting models for each trait were chosen based on Akaike Information Criterion (AIC) and model residual plots were visually checked for normality. Broad sense heritability of each trait was calculated in Genstat using the method of Cullis et al. (2006) for each best fitting model within trial years as well as across both years.

Correlations between trait BLUPs in each year and across years were calculated using the Pearson correlation coefficient. Traits from meta-analysis across years were clustered into 8 groups using Ward’s Hierarchical agglomerative clustering method using the ‘hclust’ function with the ‘ward.D2’ method in R (R Core Team 2013) based on a distance matrix derived from 2(1 − |r | ) where r represents the trait correlation matrix. Hagberg falling number was not strongly correlated to any other traits so was considered in a separate group.

A second trait clustering was performed including all trait BLUPs from each year in a single hierarchical clustering. For each of these eight groups of traits, a principal component analysis (PCA) was performed on scaled and centred (so that mean = 0 and variance = 1) trait data. Principal component (PC) weightings for each PCA group were then used as traits for further QTL analysis.

### QTL detection and calling

QTL mapping was performed for all trait BLUPs in each year, for generalised BLUPs across both years and for PCs from all PCAs (to analyse all traits across both years) in R (R Core Team 2013). To account for the genetic effects of co-segregating haplotype blocks (Brinton et al. 2020), we employed both SNP- and haplotype-based QTL mapping approaches. Haplotypes were inferred by identity-by-descent from the multiple MEL founders (Scott et al. 2020).

First, using a subset of 7269 unique SNP markers, each trait was regressed against each SNP marker in a linear model weighted by the number of lines per funnel to adjust for the minimal population structure resulting from unequal numbers of individuals per 210 MEL funnels (specific crosses made during the inter-crossing stage of population development). QTL for single marker main effects were selected based on a recursive forward selection and backwards model fitting strategy as employed by Broman and Sen (2009), as follows:

(i) Identification of the most significant peak marker as a significant main effect in the initial scan. (ii) Repeat the scan with the first peak marker included as a fixed effect. (iii) Repeat step (ii) until no main effect peaks with *p* < 0.00042 are found. This threshold for addition of QTL to the full model was based on a Bonferroni corrected alpha = 0.1 of the estimated number of independent haplotype blocks across the whole genome rather than the total number of SNPs. This was calculated to be 237.19 haplotypes considering four recombination events per chromosomes and a map length of 54.05 Morgans. For each QTL added to the model, *p* values were also adjusted for false discovery rate (FDR) using the method described by Benjamini and Hochberg (1995) and QTLs with FDR *p* < 0.05 at this stage were considered high confidence. (iv) With all main QTL effects added as fixed effects, this full model was then reduced, with marker effects being removed based on AIC using the ‘step’ function in R with *k* = 13.73 to define the final fitted model. This value of *k* was used to remove QTL with an approximate equivalent *p* > 0.00021, which was used as the Bonferroni correction with alpha = 0.05 based on total haplotype number and removed QTL at a stricter threshold than they were added in at. QTL that remained in the fitted model but were not already identified as high confidence based on FDR *p* value, were considered low confidence.

Markers flanking each QTL interval were defined as those outside but closest to the interval of markers on the physical map position that were within 20 cM from the peak and that were within two –log10 *p* from the peak marker. The percentage of phenotypic variance explained by each QTL effect was calculated from the proportion of sum of squares to the total sum of squares in each of the fitted models and the percentage of phenotypic variance explained by all QTL per trait was calculated from the adjusted R^2^ value of the fitted model.

Second, haplotype mapping was performed with founder haplotype probabilities, i.e. for every region of the genome in a RIL, the probability that the haplotype came from each founder. These were calculated from the set of 7269 SNP markers using the ‘mpprob’ function in R/mpMap (Huang and George 2011) at 1 cM step intervals. QTL mapping for this haplotype approach was performed in the same forward and backward model fitting approach as for regression on SNP markers except a value of five was used for *k* in the model reduction function using ‘step’.

Rather than setting strict thresholds for calling QTLs for each trait, across and within both experimental trial years, and using each analysis method, the importance and relevance of each QTL was assessed by identifying co-localised consensus QTLs based on the body of evidence across results from all traits, trial years and both analysis methods. Pairs of QTL were considered co-localised if they were on the same chromosome, had peak markers within 20 cM of each other and had correlated founder effects (r > 0.6). This value of genetic map distance was used to allow for potentially large D genome blocks with no detectable recombination (Gardner et al. 2016). Hierarchical cluster analysis was performed on the matrix of all QTL pairs, where co-localised pairs had a distance of 0 and distinct pairs had a distance of 1, using the ‘hclust’ function in R with the ‘single’ clustering method. Individual QTL were assigned to pleiotropic consensus QTL groups by using the ‘cutree’ function at a threshold of 0.5.

## Results

### Trait heritability and effects of flour refinement on mineral density

Milling, baking and mineral density traits were assessed in trials over two years in the UK, closely reflecting standard breeding programme trialling capacity. Ranges and distributions of all traits are shown in Supplementary Fig. S1. Broad sense heritabilities for each trait measured within and across years varied widely. Traits such as grain dimensions or some dough rheological traits (e.g. DoughLab softening and stability) had consistently high heritability (>0.8), whereas traits such as Hagberg falling number and most micronutrient concentrations, with the exception of Ca, had relatively low heritabilities (<0.5) (Table 1). Heritability across years was similar to within year values for traits such as grain protein content and specific weight that had relatively high correlations of BLUPs between the two years (0.63 and 0.82, respectively) indicating low genotype by environment (G × E) interactions. On the other hand, traits including Hagberg falling number, Se and Zn had lower heritability across years than within years, and a low correlation between BLUPs between years indicating larger G × E effects.

**Fig. 1:**
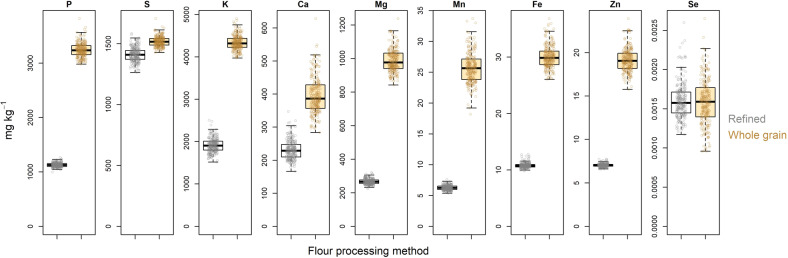
Differences in micronutrients concentrations between refined white and whole grain flour. Points represent BLUP values for 231 lines grown in field trials across two years. Horizontal lines within each box represents the median value.

Analysis of mineral densities in refined white and whole grain flour revealed large differences between the two processing methods in average micronutrients value, as well as in the variance around the averages (Fig. 1). Refinement significantly reduced P, Mg, Mn, Fe and Zn (*p* < 0.001) to approximately one third of the value of whole grain flour whilst it reduced Ca by close to a half (*p* < 0.001). In contrast, values of Se were comparable between the two milling methods and S levels were only slightly reduced (*p* < 0.001) in refined white flour.

### Relationships among milling, baking and nutritional traits

**Fig. 2:**
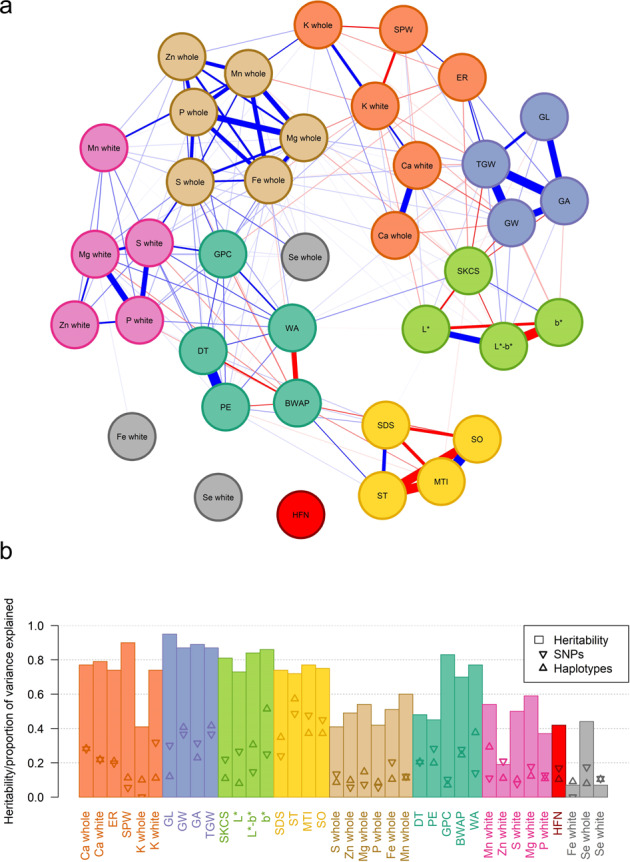
Relationships among traits and the proportion of the trait heritability explained by QTL. **a** Network analysis of all analysed milling, baking and micronutrients traits across two trial years identify eight distinct groups. Blue and red connecting lines indicate positive and negative correlations, respectively and line width is proportional to correlation strength. Only correlations with *p* < 0.001 are shown. **b** Proportion of phenotypic variation explained by the broad sense heritability as well as all QTL included in a full model for SNP and haplotype-based analysis for meta-analysis across two trial years for all traits. GPC = Grain protein content (%); SPW specific weight (kg hl-1), SKCS single kernel characterisation system hardness, ER extraction rate (%); L* = Flour whiteness (Tristimulus L*); b* = Flour yellowness (Tristimulus b*); L*-b* = overall flour colour (Tristimulus L*-b*); HFN = Hagberg Falling Number (s); GA = MARVIN grain area (mm^−1^); GL = MARVIN grain length (mm); GW = MARVIN grain width (mm); TGW = MARVIN thousand grain weight (g); BWAP = DoughLab Bandwidth at Peak; DT = DoughLab Development time (s); MTI = DoughLab mixing tolerance index; PE = DoughLab Peak Energy; SO DoughLab Softening; ST = DoughLab Stability; WA = DoughLab Water absorption (%); SDS = SDS Sedimentation (ml); whole = mineral concentration in whole meal flour (mg kg^−1^); white = mineral concentration in refined white flour (mg kg^−1^).

Correlation analysis revealed relationships among traits within and between groups (Supplementary Table S1; Fig. 2a). Positive correlations were found between traits that are considered useful predictors of harder to measure functional traits in breeding selection. For example, flour water absorption values were generally higher in lines with high grain protein content and endosperm hardness (Fig. 2a). SDS sedimentation was also found to strongly relate to dough rheology traits such as mixing tolerance index, softening and stability indicating high gluten quality (Fig. 2a). Extraction rate, which is an important but difficult to predict trait, was found to relate to several other grain morphology traits. Lines with high protein, large, high specific weight and softer grains had higher extraction rate (Fig. 2a). Grain width rather than length drove the relationship between grain size and extraction rate (Fig. 2a).

Concentrations of most micronutrients in whole grain and white flour were positively correlated with each other, with the exception of K content in white flour, which was negatively correlated with Mn, Mg and Fe in whole grain flour (Fig. 2a). Clear relationships between functional quality traits and micronutrient density traits were also detected. Most notably, grain protein content correlated positively with concentrations of most micronutrients in both whole grain and white flour with the exception of Fe, K and Ca in white flour (Fig. 2a). Other milling quality traits such as specific weight and extraction rate had negative relationships with micronutrient content in white flour, in particular with K and Ca (Fig. 2a). This likely indicates that high extraction rate flours found here have the nutritionally denser bran fractions efficiently separated from the white flour and represents a trade-off between processing efficiency and nutritional value. However, despite the positive link between both thousand grain weight and grain width with specific weight and extraction rate, the relationships between grain size traits and different micronutrients varied. For example, high thousand grain weight and high grain width lines generally had higher levels of most micronutrients in both whole grain and white flour, particularly Fe and Mg in whole grain flour, but had decreased levels of Ca and K in both white and whole grain flour (Fig. 2a). Similar to grain size traits, the flour colour trait L*-b* (indicating whiteness) had negative relationships with Ca and K in white flour but weak positive relationships with Mg and Fe in whole grain flour (Fig. 2a).

### Pleiotropic QTL effects explain trait heritability and correlations

The total amount of heritable variation that could be explained by all QTL varied among traits and analysis methods (Fig. 2b). In general, highly heritable traits were more fully explained by QTLs but relative differences in this measure reflect each trait’s underlying genetic architecture. For example, dough rheology traits, including stability, mixing tolerance index and softening had cross-year heritabilities ranging from 0.72 to 0.77, but a small number of detected QTL could explain more than half of this heritability, indicating simple genetic control with several large effects (Fig. 2b). Conversely, grain morphology traits, such as grain length, specific weight and grain protein content were highly heritable (0.83–0.95), but only a relatively small proportion of this could be explained by the detected QTL (Fig. 2b). This indicates a more complex genetic architecture involving many small and/or non-additive effects. Micronutrient concentration traits generally had low heritability, except Ca. Very low heritability was found for Fe, Se and Zn in white rather than whole grain flour, whereas the opposite was found for K and Mg (Fig. 2b). There was no clear difference in the proportion of trait variation explained between SNP or haplotype analysis methods (Fig. 2b).

**Fig. 3:**
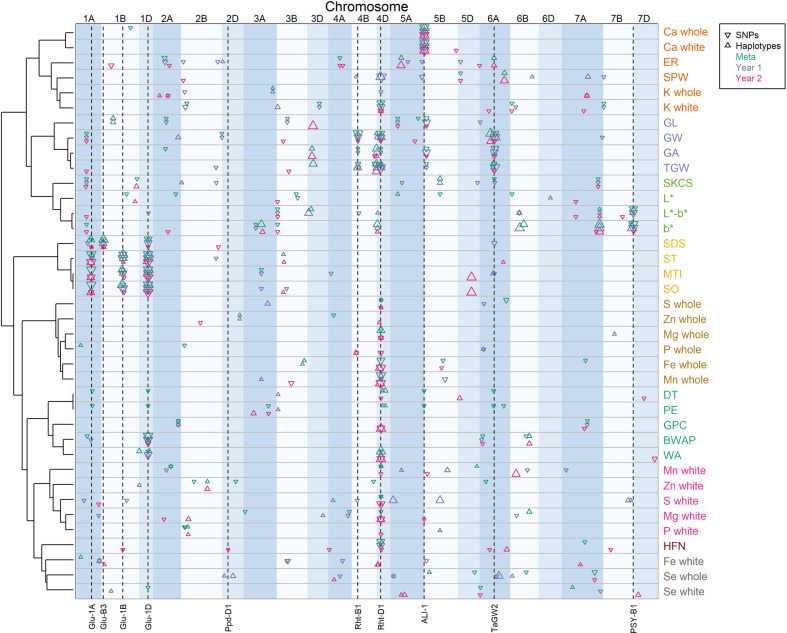
Main effect QTLs detected across all traits within each year and across both years using SNP- and haplotype-based mapping. Traits are grouped based on relationships shown in Fig. 2a. Point symbol size is proportional to QTL FDR adjusted p-value significance level. Vertical dashed lines indicate locations of known genes and QTL. GPC grain protein content (%), SPW specific weight (kg hl−1), SKCS single kernel characterisation system hardness, ER extraction rate (%); L* = Flour whiteness (Tristimulus L*); b* = Flour yellowness (Tristimulus b*); L*-b* = overall flour colour (Tristimulus L*-b*); HFN = Hagberg Falling Number (s); GA = MARVIN grain area (mm^−1^); GL = MARVIN grain length (mm); GW = MARVIN grain width (mm); TGW = MARVIN thousand grain weight (g); BWAP = DoughLab Bandwidth at Peak; DT = DoughLab Development time (s); MTI = DoughLab mixing tolerance index; PE = DoughLab Peak Energy; SO DoughLab Softening; ST = DoughLab Stability; WA = DoughLab Water absorption (%); SDS = SDS Sedimentation (ml); whole = mineral concentration in whole meal flour (mg kg^−1^); white = mineral concentration in refined white flour (mg kg^−1^).

A total of 280 independent consensus QTL across the genome were identified across all traits and analysis methods (Supplementary Table S2). Comparison of analyses across traits provided further evidence for pleiotropic genetic effects, as trait correlations shown in Fig. 2a were mostly explained by pleiotropic QTL effects in common between correlated traits (Fig. 3).

### Genetic control of functional quality traits

Large effects of well-known genes and QTLs were detected for most of the functional milling and baking quality traits. QTL peaks located at the three homoeologous HMW glutenin genes on chromosomes 1A, 1B and 1D (Fig. 3) explained a large proportion of the phenotypic variation in dough rheology traits where the *Glu-1D* loci had the largest effect (explaining ~40% of the phenotypic variance in stability, softening, mixing tolerance index in year 1 and 15% in year 2 based on SNP analysis). Three of the four bread making quality founders (37.5% of all eight founders), and 37.2% of the lines included in the study, carried the 5 + 10 rather than the 2 + 12 or 3 + 12 HMW subunit at the *Glu-1D* loci (Supplementary Table S3). The 5 + 10 allele increased dough stability from 4.4 to 6.3 and decreased softening from 63.5 to 43.0. A QTL in the telomeric region on the short arm of chromosome 1B also co-located with the known low-molecular weight glutenin gene (*Glu-B3*) for SDS sedimentation but was not found for more specific dough rheology traits (Fig. 3). Other smaller effect QTL were found for these traits but were inconsistent between trial years or analysis methods. However, of note, a highly significant QTL on chromosome 5D was found only through haplotype analysis in year 2 for softening and mixing tolerance index but not for the closely related stability trait (Fig. 3).

Similarly to traits relating to gluten quality, gluten quantity traits were affected by the *Glu-1D* locus but were largely influenced by the *Rht-D1b* allele on chromosome 4D (Fig. 3). Lines carrying the reduced height allele had 0.22 and 0.65 percentage points lower grain protein content in year 1 and 2 respectively, and lower water absorption by one percentage point, but also a faster development time by ~13 sec analysed across both trial years. Several other small effect QTLs were found but were inconsistent across years, with the exception of a QTL on chromosome 7A which was found for grain protein content in both trial year analyses as well as the meta-analysis for SNP associations but not for haplotypes (Fig. 3). This QTL explained over 6% of the phenotypic variation in each year and the positive marker allele carried by half of the founders (Brompton, Hereward, Rialto and Xi-19) increased protein content by 0.3 and 0.5 percentage points in each trial year, respectively.

Grain morphology traits were also found to be under strong pleiotropic genetic control. *Rht-B1b* and *Rht-D1b* alleles on chromosomes 4B and 4D consistently reduced grain size (thousand grain weight, grain area and grain width) (Fig. 3). Similarly, a QTL on chromosome 6A associated with the known *TaGW2* gene explained a large proportion of the phenotypic variation for these traits (Fig. 3). Grain length remained an exception to the other grain morphology traits and independent genetic control of grain width and length was clear with no QTL in common. Instead, a QTL on chromosome 5B had consistent positive pleiotropic effects on grain length as well as thousand grain weight and grain area (Fig. 3), which explained 10.6 and 5.9 percent of the variation in grain length in year 1 and 2, respectively. Another QTL on chromosome 5 A was found in common between the two trial years for grain length but the effect was smaller and not in common with other grain morphology traits (Fig. 3). Specific weight was more closely related to the milling quality trait extraction rate than the specific grain morphology traits and had more pleiotropic QTL in common. Although *TaGW2* was found to also increase extraction rate in line with increasing grain width, both *Rht-B1b* and *Rht-D1b* height reducing alleles, which reduced grain size, did not affect extraction rate in the same way. Other more minor and inconsistent QTL were found that explain the association between specific weight and extraction rate. These include QTL on 2B and 5 A identified through meta-analysis using SNP associations of specific weight that both also had pleiotropic effects on extraction rate in year 1, and a QTL on 5D that was in common between the two traits but identified in different years through SNP analysis only.

Multiple small effect QTL were found consistently across trial years and analysis methods for different related flour colour traits (b*, L* and L*-b*). However, the known *Psy-B1* gene on chromosome 7B explained the greatest percent of the phenotypic variation in b* (14.1 and 6.9% in years 1 and 2, respectively) and L-b* (10.7% in year 2 only). The link between both b* and L* flour colour traits and grain hardness was confirmed by a QTL on chromosomes 1A in common for b* and grain hardness in year 2, (explaining 6.0% and 5.6% of the phenotypic variation in the two traits, respectively), and the allele inherited from the founders Brompton, Soissons and Xi-19 had greater b* by 0.37 and SKCS hardness by 5.2. Other small effect QTL had overlapping intervals between the two traits, but uncorrelated founder effects so were not considered pleiotropic.

Values of Hagberg falling number were higher in year 1 (mean = 378) than year 2 (mean = 294) due to much lower precipitation before harvest in July in year 1 (23.2 mm) than year 2 (42.9 mm) (rainfall data from the nearest Met Office historic weather station; Oxford: Lat = 51.761 Lon = −1.262; Supplementary Table S4). Hagberg falling number was not found to be strongly correlated with other analysed traits and only *Rht-D1* was found as a pleiotropic QTL for this trait where the height reducing *Rht-D1b* allele increased Hagberg falling number by 21.4 and 13.9 sec in years 1 and 2, respectively. No other QTL were consistent between years or analysis methods for Hagberg falling number. However, a QTL co-locating with the photoperiod insensitivity allele *Ppd-D1a* inherited from the only insensitive founder ‘Soissons’ was found on chromosome 2D only through SNP analysis in year 2. Lines with the insensitive allele had lower Hagberg falling number by 18.3 sec in year 2 when Hagberg falling number values were on average lower, whereas in year 1 insensitive lines had greater Hagberg falling number by 10.9 sec.

### Genetic control of micronutrients traits

Genetic control of mineral micronutrients concentrations in whole grain and white flour was also found to be determined by several QTL that explained the strong correlations among and between nutrients and other functional quality traits. As well as having effects on many grain morphological and protein quantity traits, *Rht-D1* was found to have wide-ranging effects on mineral content in both whole grain and white flour. Considered across both trial years, the height reducing *Rht-D1b* allele decreased concentrations of Mn (−7.3%), Fe (−4.6%), P (−1.8%), Mg (−4.2%), Zn (−2%), Se (−8.7%) and S (−1.7%) in whole grain flour, as well as of Mn (−3.3%), P (−1.5%), Mg (−3.2%) and S (−2.5%) in white flour. However, the opposite effect was found for K where the reduced height allele increased concentrations by 1.9% and 6.8% in whole grain and white flour, respectively. A large effect and highly significant QTL, that co-located to the awn length inhibitor locus (*ALI-1*; Wang et al. 2020) on chromosome 5 A, was found for Ca in both whole grain and white flour in all analysis methods. The single locus explained 23.9% and 22.2% of the phenotypic variation in whole grain and white flour, respectively, where lines with awns, inherited from the only awned founder (Soissons), had 24.0% and 20.4% higher Ca content than non-awned lines for whole grain and white flour, respectively.

Other QTL for micronutrients were less consistent but linked nutritional traits to functional quality traits. For example, although the correlations between gluten quality traits and micronutrients were small, the positive effect on dough rheology traits (stability, softening and mixing tolerance index) at the *Glu-1D* locus was also found to increase Fe in white flour. A QTL that had a positive effect on mixing tolerance index and softening on chromosome 3 A was also found to increase Mn (in the haplotype analysis) in whole grain flour. However, relatively few other QTL could explain the close correlations among nutrients in whole grain or white flour, possibly due to the low heritability of these traits and strong environmental effects. One QTL on 6 A was found for both P and S in whole grain flour and a group of possibly co-locating QTL on 5B for Fe found in both year 2 and meta-analyses and Mn in whole grain flour (all using SNP analysis). For micronutrient traits in white flour, a group of overlapping QTLs on chromosome 6B was found for Mg and Mn in white flour, although their founder effects were not exactly synchronous between traits and trial years. Another QTL on chromosome 2B was found to have pleiotropic effects on both P and Mg in white flour through haplotype analysis.

### PCA multi-trait analysis detects novel genetic effects to optimise trait trade-offs

**Fig. 4:**
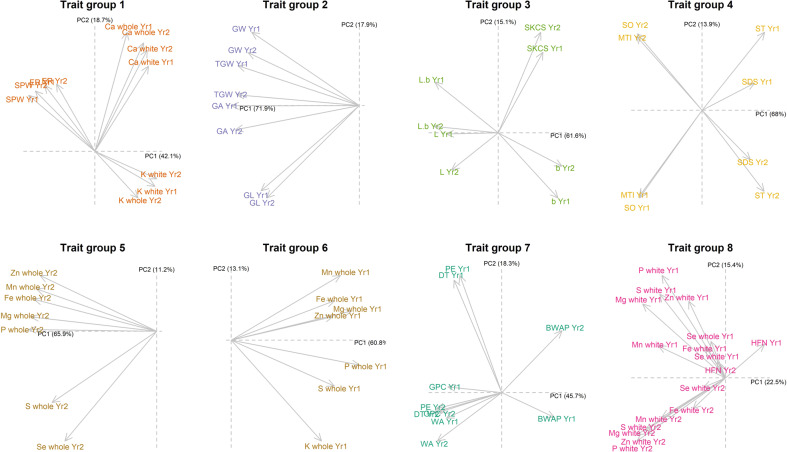
Weightings of the first two principal components for each trait group. Trait groups were defined by hierarchical clustering for traits measured in each trial year. Arrows indicate the direction of trait weightings where traits with similar weightings for PC1 on the *x* axes are positively correlated. PC2 on the *y* axes show an example of higher order PCs that contrast effects that are orthogonal to PC1 overall trait correlations.

For PCA based QTL mapping, traits measured within each trial year were grouped into eight groups by hierarchical clustering (Fig. 4). Similarly to groupings for meta-analysis traits across both years (Fig. 2b), group 1 traits included Ca and K mineral density traits with milling quality traits (extraction rate and specific weight), group 2 traits included grain dimensions and morphology traits, group 3 traits included endosperm texture and flour colour traits, and group 4 traits included gluten quality traits (Fig. 4). Conversely to groupings for meta-analysis (Fig. 2b), micronutrient traits measured in whole grain flour were grouped into two separate groups for each trial year (groups 5 and 6) (Fig. 4). Group 7 included several dough rheology traits relating to gluten quantity and flour water absorption, while group 8 included many micronutrient density traits measured in white flour, that were measured with lower heritability, and Hagberg falling number which had low correlations with other traits (Fig. 4).

**Fig. 5:**
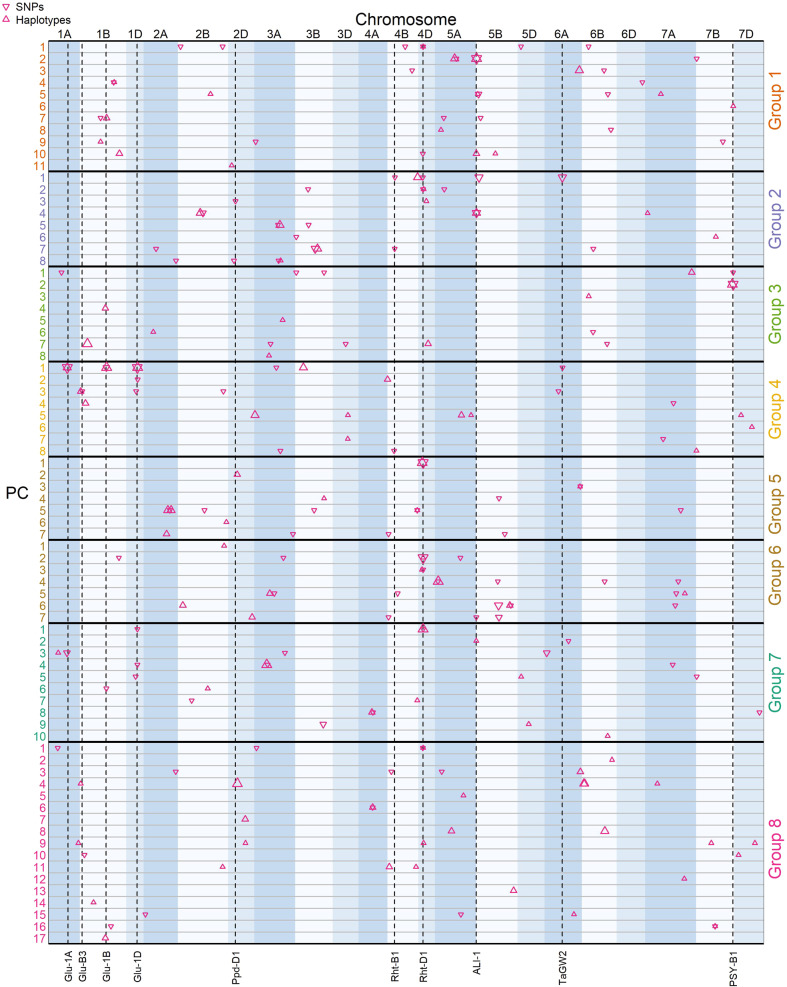
Main effect QTL locations found across all principal components multi-trait analyses for all trait groups using SNP based and haplotype-based mapping methods. Traits measured separately for each trial year were grouped and analysed in separate principal component analyses. Point symbol size is proportional to QTL FDR adjusted *p* value significance level. Vertical dashed lines indicate locations of known genes and QTL.

**Fig. 6:**
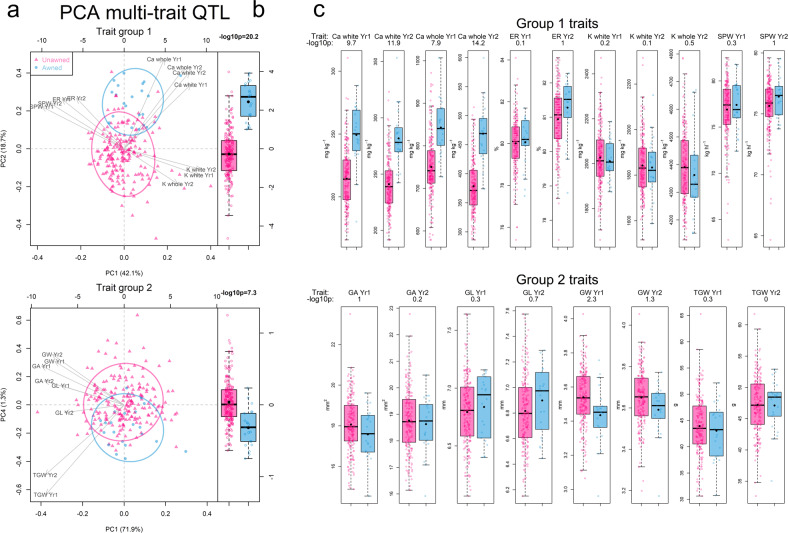
The pleiotropic genetic effects of the awns locus (ALI-1) identified through PCA multi-trait QTL analysis in two groups of correlated traits. **a** PCA biplots to the left include trait and line weightings on the first PC on the *x* axis and the PC for which PC QTL were identified on the *y* axis. Non-awned and awned lines are indicated by pink and blue colours, respectively, across all plots. The percent of the phenotypic variance explained by each PC across all traits in each group is given in parenthesis in axis labels. **b** Boxplots alongside the biplots indicate the genetic effect of the awns PCA QTL on the *y* axis PC. **c** Boxplots to the right also indicate the genetic effect of awns for each trait within each group. Horizontal lines within each box represent the median and black points represent the mean values. –log10p values of QTL effects are shown above each plot. SPW specific weight (kg hl^−1^), ER extraction rate (%); GA = MARVIN grain area (mm^2^); GL = MARVIN grain length (mm); GW = MARVIN grain width (mm); TGW = MARVIN thousand grain weight (g); whole = mineral concentration in whole meal flour (mg kg^−1^); white = mineral concentration in refined white flour (mg kg^−1^). Yr1 and Yr2 indicate traits measured in year 1 and 2, respectively.

PCA based QTL mapping for all PC of each trait group identified additional genetic effects that contrast between traits (Fig. 5; Supplementary Table S2). Of the 155 consensus QTL identified from the PCA method, 101 were unique and not found using single trait analysis. Some of these demonstrate interesting and novel genetic effects that are orthogonal to the overall trait correlations. As an example of a PCA QTL that optimised a trade-off between functional and nutritional quality traits, the second PC of the group 1 traits had positive weightings for both extraction rate and Ca in white flour, two traits which were negatively correlated (r = −0.2 across years) (Fig. 2a), and therefore had opposing weightings for the first PC (Fig. 6). The genetic effect of awns (controlled by the *ALI-1* gene) increased Ca concentration in whole and white flour (as outlined above), but also a slight increase in average extraction rate and specific weight (Fig. 6), despite their negative correlation with Ca (Fig. 2b). Among the closely positively correlated group 2 (grain morphology) traits, the awns locus was detected with high confidence for PC4 (Fig. 6). Awned lines had similar thousand grain weight despite decreasing other closely related grain dimension traits (grain area and width). QTL effects from the awns locus were far too small to be detected for any of the group 2 traits from single trait analysis, but while the PC4 only explained 1.3% of the phenotypic variance across traits in the group, 19.3% of the variance in PC4 could be explained by the highly significant awns QTL. This example therefore indicates how multi-trait PC QTL analysis greatly increases the power to detect small effect QTL by considering novel pleiotropic effects across related traits.

Other novel PCA QTL include one on chromosome 5B that was not found from any single trait analysis (Fig. 5), where the minor allele inherited only from the founder Xi-19 had a negative effect on extraction rate (−0.27 percentage points) and a positive effect on specific weight (0.9 kg hl^−1^) across years, despite the positive correlation of these traits (Fig. 2a). The role of the *Psy-B1* gene, identified for flour colour traits in the single trait analysis, was also identified with higher confidence in the second rather than first PC of the group 4 traits (Fig. 5), where its effect was to reduce flour yellowness (b*) (−0.50) without the associated decrease in grain hardness, despite the two traits’ positive correlation (Fig. 2a).

Genetic effects that contrast among nutritional traits were also identified. P was found to correlate positively with most other important mineral nutrients, including Fe, Mg, Mn and Zn (Fig. 2a), and is known to be largely stored in the grain as phytic acid, which is known to have anti-nutritional effects on bioavailability of associated mineral elements (Gupta et al. 2015). Therefore, PC QTL that can increase mineral nutrient density without associated P would have the potential to increase the concentrations of key nutrients such as Fe, Mg, Mn or Zn in more bioavailable forms. Two independent QTL on chromosome 5B were found for group 5 (year 2) and 6 (year 1) whole grain flour nutritional traits (Fig. 5), that each increased Mg in whole grain flour by 16.8 mg kg^−1^ in year 2, or 20.7 mg kg^−1^ in year 1, while slightly decreasing P concentration. Additionally, a QTL that co-located with *Rht-D1* was found for trait group 6 PC2 (Fig. 5), where the dwarf allele reduced Mn in whole grain flour in year 1 by −1.7 mg kg^−1^ (−7.1%), whilst only also slightly reducing P by −14.5 mg kg^−1^ (−0.47%).

### PCA multi-trait analysis detects novel G × E effects

Although the present study included only two trial environments, our results suggest that considering the same trait measured in different trial years as separate independent traits in the PCA QTL analysis also meant that QTL effects involved in G × E interactions could be identified. Critically, these were not identified in either trial year alone. As an example, the effect of the photoperiod sensitivity (*Ppd-D1*) locus on chromosome 2D was found for group 2 PC3 (Fig. 5), which highlighted the differential effect of *Ppd-D1* on grain morphology traits between the two trial years. All grain morphology traits in year 1 were positively weighted on PC3 whereas equivalent traits in the second year were weighted negatively. While thousand grain weight correlated well between the two years (r = 0.78), lines that carried the insensitive *Ppd-D1a* allele, inherited from the founder Soissons, had slightly higher thousand grain weight in the second year (0.21 g) but lower in the first year (−0.26 g) when thousand grain weight values were on average lower, suggesting reduced stability across the two environments.

## Discussion

Genetic control of milling, baking and nutritional traits was analysed in a wheat MAGIC population over two trial years reflecting current practice for selection in applied wheat breeding. A large number of QTL were identified, which confirmed major effects of known loci in addition to detecting novel QTLs of smaller effect. This is consistent with indications that few large effect genetic effects remain to be found that further explain trait variation (Scott et al. 2021). Many of the micronutrient traits were found to be of low heritability or inconsistent between trial years, suggesting limited potential for direct selection in breeding. However, it may be more effective in crop breeding to practice indirect selection on related traits with greater heritability and consider genetic effects that optimise the interplay among related traits. We found that pleiotropic QTL effects could explain many of the correlations among traits and that a PCA-based approach to QTL mapping identified additional QTL with novel antagonistic effects that could be used to optimise trait trade-offs.

Major findings from this study were the identification of the role of the *Rht-D1b* dwarfing allele on many functional quality traits, and its negative effect on grain mineral concentrations, and the previously unreported, large and consistent positive effect of awn presence on Ca concentration in both whole grain and white flour. We further present evidence that genetic effects contrasting between correlated grain micronutrients may be used to increase the ratios of important micronutrients, such as Mn, and P, thus increasing the concentration of micronutrients in a more bioavailable form (Gutpa et al. 2015).

Replication of a subset of lines within each trial year and almost all lines across the two trial years enabled estimation and comparison of trait heritabilities. While most traits were highly heritable across years, some of the micronutrient density traits, such as Fe content and flour whiteness, particularly in year 2, were lower due to contamination of grain with extraneous soil and dust particles from harvesting field grown plots. Some dough rheology traits, including bandwidth at peak, development time and peak energy, had much lower heritability in year 1 whena second and larger peak in the development curve was sometimes found, similarly to Bason et al. (2005). Heritability of Hagberg falling number was considerably lower across than within years which reflects the strong environmental component to this this trait and the contrasting weather patterns between the two trial years (Smith and Gooding 1999). Similar to results reported by Scott et al. (2021) in a 16 founder MAGIC population, the proportion of the heritable trait variation that could be explained by detected QTLs was dependent on the complexity of trait genetic architecture. Additional small genetic effects may explain the remaining heritability but were not detected using the statistical power available in this study. Wright et al. (2021) investigated similar traits in the same population with a similar number of lines and found that the probability of detecting small effect QTL (explaining <10% of the variation) was very low, unless the trait heritability was very high. We acknowledge that low heritability of some traits and testing in only two trial years may limit the extent to which results can be interpreted. This highlights the difficulty in detecting QTL with genome-wide significance for highly polygenic traits, even in highly recombined MAGIC populations (Scott et al. 2021). Methods that integrate cross-phenotype associations are widely found provide additional information to genetic association studies and increase the power to detect QTL (Mortezaei and Tavallaei 2021). Therefore, rather than setting a strict statistical significance threshold for detection of QTL, we considered co-localising QTL across related traits, trial years and analysis methods to assess biological significance. We were therefore able to show that pleiotropy of detected QTL was responsible for correlations between related functional quality traits as well as negative trade-offs, such as between functional milling and nutritional quality traits. PCA based QTL analysis, which identifies orthogonal contrasts among the multivariate trait data, was then used to further determine genetic effects that could be used to optimise negative trade-offs. The first PC of each group captured the greatest proportion of phenotypic variation so that correlated traits have similar PC weightings. However, higher order PCs capture variation that contrasts the overall trait correlations where traits are weighted together despite being negatively correlated and so represent genetic effects that are often not evident from single trait analyses.

### Application of milling and baking quality trait QTL for wheat breeding

For traits relating to functional milling and baking quality, most large effect QTL could be related to known genes or QTL. The dough rheology traits were largely controlled by the well characterised HMW glutenin genes (Payne and Lawrence 1983). Their effects and the favourable combinations of the three homoeologous loci are well-known and readily selected for in milling wheat breeding programmes in the UK (Liu et al. 2012). Few other genetic effects that were consistent between the two years were found which might explain the relatively small amount of remaining heritability for these traits. This is consistent with the range of end use classes of the population founders (Supplementary Table S3) and the strong selection by breeders for consistency of bread quality traits. However, the founder variety Hereward is an exception as it is known to have high quality despite a poor profile of HMW glutenin genes (Shewry et al. 1992), but its alternative genetic control of high quality was not identified here. Only 17 (7.4%) of the lines did not carry the beneficial 5 + 10 allele at the *Glu-D1* locus and had greater dough stability than Hereward. A QTL from only one of the eight founders would need to be of large effect to be detected in the population suggesting complex genetic control by many small and possibly epistatic genetic effects explains Hereward’s high functional gluten quality. Hereward is also the oldest of the eight founders and had the greatest protein content but lowest dough stability compared to the other high bread making quality founders (Rialto, Soissons and Xi-19), reflecting recent trends in breeding stronger gluten but lower protein content wheat varieties (Call et al. 2020).

Wheat with low Hagberg falling number (below 250) due to excessive alpha amylase activity often results in poor loaf volume and structure (Perten 1964). The *Rht-D1b* dwarfing allele was found to have a consistent and large positive effect on Hagberg falling number, which is supported by similar results from other studies (Börner et al. 2018; Gooding et al. 2012). Mares and Mrva (2014) suggest that the effect of the dwarfing genes on late maturity alpha amylase, which is known to reduce Hagberg falling number in the absence of pre-harvest sprouting, is due to a direct effect of gibberellic acid. However, *TaGW2* is known to similarly regulate gibberellic acid synthesis (Li et al. 2017) and grain size but, unlike *Rht-D1b*, was not found to significantly affect Hagberg falling number in the present study, suggesting independent pleiotropic effects. The majority of current varieties in the UK possess *Rht-D1b* (Bentley et al. 2014) so its beneficial effect is generally already selected for in breeding programmes. As well as the known effect of *Rht-D1b*, we found that the photoperiod insensitive allele *Ppd-D1a*, despite low frequency in the population (11.3%), reduced Hagberg falling number in the year when Hagberg falling number values were generally lower due to above average rainfall before harvest. Although this suggests that insensitive lines have less stable Hagberg falling number, this effect may be due to the practicalities of harvesting material with varying maturities. Agronomically, earliness is valued to enable ease of crop management logistics allowing earlier harvest before potential adverse weather conditions later in the season (Sheehan and Bentley 2021). No other consistent QTL were found for Hagberg falling number. However, a QTL on the short arm of chromosome 6A had positive founder effects matching almost exactly with all the high grain quality founders (Hereward, Soissons and Xi-19) with the exception of Rialto, which is known to be susceptible to pre-maturity alpha-amylase activity (Tjin-Wong-Joe 2004). The lack of large effect QTL for Hagberg falling number reflects its relatively low heritability and large G × E effects. Kristensen et al. (2018) also found that variation in Hagberg falling number could not be explained easily by single marker effects.

Several small effect QTL for extraction rate and specific weight explained only a small proportion of the trait heritabilities, highlighting their complex nature. The previously reported large differences in extraction rate between hard and soft wheats controlled by the puroindoline genes (Campbell et al. 2001) were fixed in the lines assessed here. Further genetic markers controlling extraction rate, a complex, costly and important milling efficiency trait, have remained largely elusive as genetic markers for this trait have yet to be routinely employed in breeding. However, considering pleiotropic effects between these traits and other related grain morphology traits sheds some light on their component phenotypes and provided stronger evidence for QTL than traits considered independently. While both Rht genes were found as high confidence QTL for grain size traits (grain width, area and thousand grain weight) but not extraction rate, a positive pleiotropic effect of the *TaGW2* locus in increasing extraction rate along with increased grain size further reinforces the value of this already well characterised gene (Simmonds et al. 2016). Small effect QTL on 2B, 5A and 5D were also found in common between specific weight and extraction rate and PCA based QTL mapping found a QTL on 5B that had contrasting effects between the two traits. While these few novel QTL effects for Hagberg falling number and extraction rate may prove valuable for marker assisted breeding, our approach of assessing genetic effects for multiple traits may be more effective in optimising traits under complex pleiotropic control.

### Resolving trade-offs with grain nutritional quality

Our findings also shed light on the genetic basis of the mineral concentration dilution effect (Davis 2009; Fan et al. 2008), as well as trade-offs between grain nutritional content and functional quality traits. In general, micronutrient concentrations were strongly associated with protein content which supports findings in other studies (Cakmak et al. 2010). This trend is likely to be related to the close trade-off and negative correlation between yield and protein (Michel et al. 2019; Simmonds 1995). Supporting findings by Jobson et al. (2018), our results show that the height reducing allele *Rht-D1b* had the largest effect on both nutrient density and protein content. This locus underpins the high yielding nature of post-green revolution wheats (Hedden 2003). The reduced height allele is present in the majority of current UK wheat varieties but we suggest that this only partly accounts for the observed dilution. The amount that *Rht-D1b* reduced nutrient density (generally < 5% for most elements) is less than the decreases found by Fan et al. (2008) in recent decades (20–30%), suggesting that many other small genetic effects have enabled recent yield increases but have also contributed to the dilution effect. Whilst other small effect QTL in a limited number of environments were identified here that could be used to implement selection for biofortification, caution should be taken when selecting for QTL identified for micronutrient density where opposing negative effects on yield are likely to be present. Increasing grain yields at the expense of nutritional value has been problematic, but the opposite approach of increasing nutritional value by lowering yield would be no better considering calorie requirements of a growing population. Regarding the link between mineral concentration and protein content, we suggest that selection on an index of high grain yield-protein deviation, that breaks the trade-off between yield and protein content (Michel et al. 2019), would also have indirect effects on micronutrient density. Based on evidence in literature, this has not yet been implemented in wheat breeding. Despite protein being a valuable quality trait that is selected for in bread wheat varieties, protein content levels have been shown to have decreased in more modern varieties where sufficient baking performance has instead been achieved by increasing the ratio of glutenin to gliadin proteins and resulting gluten strength (Shewry et al. 2020). However, a suitably high heritability for the grain yield-protein deviation has been found (Mosleth et al. 2020), which suggests that positive genetic gain would be possible using appropriate selection indices and genomic prediction tools (Michel et al. 2019; Scott et al. 2021). This approach is thought to also result in increased nitrogen use efficiency (Fradgley et al. 2021).

Our results showed that, as expected, there were generally much greater concentrations of micronutrients in whole grain flour before refinement due to their greater concentrations in the bran fractions (Slavin et al. 2000). In addition, we found that there was also greater phenotypic variation and clearer QTL effects in whole grain flour than white flour. Capitalising on the already well-established health benefits of whole grain cereals and increased intake of whole grain bread would have immediate positive impact (Poole et al. 2020). Whilst it has been suggested that the nutritional value of white flour, with respect to insoluble fibre from starch, has increased (Lovegrove et al. 2020), this has been at the expense of amino acid and mineral micronutrient concentrations (Shewry et al. 2016). Genetic improvement in nutritional components that have been diluted by increased starch content within the narrow range of phenotypic variation in white flour will be difficult given the limited genetic effects in the current wheat gene pool and the specifications of the milling and baking industry. Murphy et al. (2008) suggested that decreases in mineral content of soft white spring wheat cultivars were due to breeders’ selection for low flour ash content. While ash content is not commonly used as a quality evaluation in the UK, as it is in the US, we found that high extraction rate and bright white flour colour were also negatively associated with some mineral concentrations. This suggests that continued breeding for these functional quality traits has further eroded the nutritional value of bread made from refined white flour, and presents even greater challenges to selecting for enhanced wheat nutritional quality within the parameters of current milling quality requirements. However, by considering pleiotropic effects across traits, we demonstrate that these trade-offs can also be optimised.

### Selection for awns: an opportunity to increase grain Ca content

We detected a large and highly significant effect of presence of awns on increasing grain Ca content and grain density. This is an ideal example of a single genetic effect that could be used to optimise the trade-off between nutritional density and milling quality traits. The only awned founder of this population was Soissons and a minority of current UK and European milling wheat varieties are awned (Würschum et al. 2020). Therefore, the beneficial effect of awns could easily be selected for phenotypically within the current elite and high grain quality gene pool. Whilst the causative underlying awn length inhibitor 1 gene (*ALI-1*) in bread wheat has recently been identified (Wang et al. 2020), the novel effect on Ca has only recently been identified in a more limited set of varieties (Pongrac et al. 2020). Considering the high QTL significance, narrow interval, the lack of effects of awns on other mineral nutrients and founder effects matching exactly to the only awned founder, a direct effect of the *ALI-1* gene, which encodes a C_2_H_2_ zinc finger transcription factor, on Ca transport is likely rather than a closely linked independent gene also inherited from only Soissons. A link between the same *ALI-1* and greater grain yield without the associated negative effect on protein has also recently been found (Scott et al. 2021). We hypothesise that because Ca is known to be upwardly mobile in the xylem but not in the phloem (Kerton et al. 2009), increased transpiration due to photosynthetically active awns during grain filling drives Ca transport to the ear and increases deposition in the grain. The variation in values in Ca content observed in material presented here (white flour: mean = 231 mg/kg, standard deviation = 29 mg/kg; whole grain flour: mean = 396 mg/kg, standard deviation = 55.6 mg/kg) and the effect size of the largest QTL effect of awns (white flour 45 mg/kg; whole grain flour 92 mg/kg) is far less than the statutory minimum requirements of 2350 mg/kg of CaCO_3_ that flour is fortified with in the UK (The Bread and Flour Regulations 1998). However, the strategy of biofortification is considered advantageous over supplemental fortification of food products (Yadav et al. 2020).

### Balancing grain mineral content to increase nutrient bioavailability

Bioavailability of key mineral nutrients such as Fe, Zn, Mg and Mn are known to be mediated by anti-nutritional chelating effects of phytic acid (Gupta et al. 2015), which is the main storage form of P in the grain. Therefore, P concentrations can be used as a proxy for phytic acid content (Fatiukha et al. 2020). These mineral nutrients were all closely correlated with P and S suggesting that genetic improvement of mineral concentrations in a bioavailable form would be difficult. Further to the effects of *Rht-D1b* in reducing concentrations of several micronutrients, it is of additional concern that *Rht-D1b* also decreased Mn relative to P concentration in whole grain flour, which is consistent with evidence from Fan et al. (2008) who found decreases in the ratios of Fe and Zn to phytate P in wheat varieties over time. However, PCA based multi-trait QTL mapping presented here identified genetic effects that could potentially increase ratios of Mg to P or Mn to P as advocated by approaches to develop low-phytic acid crop varieties (Raboy 2020). On the other hand, there may be valuable health benefits of phytic acid in human nutrition including prevention of several cancers and diabetes (Abdulwaliyu et al. 2019). Many other factors influence the chelating effects of phytate and wheat nutritional availability such as other dietary factors (Lopez et al. 2002), and processing (Rodriguez-Ramiro et al. 2017) need to also be considered.

## Conclusions

Increased predictability of milling and baking quality traits can aid development and breeding of productive and sustainable milling wheat varieties. Few undetected large effect QTL remain in European wheat germplasm to further explain trait variation of functional quality traits. However, multi-trait PCA based QTL mapping increases the power to detect novel small effect pleiotropic genetic effects across related traits and may be used to optimise trade-offs between crop quality traits with grain nutritional content. Wheat breeding programmes should consider these approaches to achieve enhanced health impacts at the same time as increasing functional quality and productivity traits.

### Data archiving

Germplasm used in this study are available from https://www.niab.com/research/agricultural-crop-research/resources/niab-magic-population-resources. The phenotypic dataset is available on Figshare.com (10.6084/m9.figshare.18397244). Statistical methods and software used are outlined in the ‘Materials and Methods’.

## Supplementary information

Supplementary Text and Figures

Supplementary table
