# Supplementary material for: Trade-offs in the genetic control of functional and nutritional quality traits in UK winter wheat
Source: Heredity (Edinb). 2022 Apr 7;128(6):420–33. doi: 10.1038/s41437-022-00503-7 (PMC9178040; doi:10.1038/s41437-022-00503-7)

Supplementary Text 1. Details of field trial experiments.

Field trials were conducted at the DSV UK breeding station in Oxfordshire, United Kingdom (lat: 52.107428, long: -1.2855828) in the 2017-2018 and 2018-2019 growing seasons (hereafter year 1 and year 2). Soil type was a medium ironstone and texture was sandy loam. Plot dimensions were 1 x 1.2m. Trials were sown on the 5th October (year 1) and 27th September (year 2). Trial plots were in a partially replicated nested block design with the eight MEL founders and 13 of the selected lines replicated twice and the remaining 218 selected lines in unreplicated plots in year 1. In year 2, the founders and 16 selected lines were replicated twice and 204 lines were in unreplicated plots. Almost all lines (220) lines were included in both trial year experiments. There were 13 main blocks of 20 plots in both years, and year 2 included two sub-blocks of ten plots within each main block so that replicated lines were distributed evenly among main and sub-blocks. Plots were managed according to field standard inputs for milling wheat which included a total of 200 kg ha^{-1} of nitrogen (N) applied over three applications (50, 100 and 50 kg N ha^{-1} respectively), and 89 kg SO_3 ha^{-1} with the first N timing. Plots were harvested with a small plot combine and grain samples were used for subsequent phenotyping.

Supplementary Text 2. Details of end use quality phenotyping methods.

Milling and baking quality traits

Grain morphology was assessed using a MARVIN seed analyser (GTA Sensorik GmbH) using 300 – 400 grains per sample to measure average grain length (GL), width (GW), area (GA) and estimated thousand grain weight (TGW). Grain protein content (GPC) was determined by Near-Infrared Reflectance (NIR) on whole grains using a Foss, Infratec™ NOVA (AAAC approved method 39-25.01; Corbellini and Canavera, 1994). Grain sub-samples were then milled with a Foss, Cyclotec ™ 1093 lab mill and approximately 7g of flour (adjusted for flour moisture content) assessed for Hagberg Falling Number (HFN) (Perten, 1964) to determine alpha-amylase activity (AACC approved method 56-81.03). HFN was measured using a Calibre, Perten falling number 1700 instrument as the time (in seconds) taken for the stirrer to fall a measured distance through a hot aqueous flour meal gel undergoing liquefaction. Endosperm texture (hardness) was determined using Single-Kernel Characterization System (SKCS) (AACC approved method 55-31.01). Refined white flour was milled using a Laboratory Buhler roller mill (AACC approved method 26-21.02) and extraction rate (ER) was measured as the percentage of standardised white flour fraction yielded from the total grain milled. Flour colour, including brightness (L*), yellowness (b*) and overall derived colour quality (L*-b*) was measured with a reflectance colorimeter (AACC approved method 14-22.01). The Specific weight (SPW) of grain was determined using the AACC approved method 55-10.01. High-speed mixing rheology traits were measured using a Calibre, DoughLab 2500 following the AACC approved method 54-70.01. This method measures resistance of a dough to mixing and gives dough quality measures from each sample resistance curve over 10 minutes. The traits assessed include water absorption (WA), dough development time (DT), stability (ST), softening (SO), mixing tolerance index (MTI), peak energy (PE) and bandwidth at peak (BP). SDS sedimentation volume (SDS) was measured on white flour (AACC approved method 56-61.01).

Mineral nutrient composition

Concentrations of key mineral nutrients were determined for both wholemeal and white flour samples. White flour samples were sub-sampled from those milled using a Laboratory Buhler roller mill and whole grain samples were milled from approximately 200 grains of each plot using a small laboratory hammer mill that retained all of the milled flour. Between 200 and 300 mg of milled flour (+/- 1 mg) per sample was digested in concentrated Nitric acid (3 ml) and Hydrogen Peroxide (2 ml) and diluted in 14 ml of deionised water. Inductively Coupled Plasma Mass Spectrometry (ICP-MS) was then conducted to quantify concentrations of elements (Ca, Fe, K, Mg, Mn, P, S, Se and Zn) using the same methods outlined by Joy et al. (2017). Blanks and certified reference material samples were used as controls across analysis batches. After removing anomalous values for each element, exogenous Fe contamination from soil dust on the grain was corrected for by adjusting values based on the amount of Vanadium (V) in each sample. This was done by linearly regressing Fe against V and the residual values from which added to the intercept were the adjusted Fe values. Mineral concentrations (in mg kg^{-1}) of soil samples bulked across each trial and analysed using ETPA extraction were Mn = 18.64, Fe = 30.11, Co = 0.09, Ni = 0.57,

Cu = 0.55, Zn = 0.94, Cd = 0.03 and Pb = 1.49 in year 1, and Mn = 33.42 , Fe = 55.13, Co = 0.16, Ni = 0.92, Cu = 0.78, Zn = 1.21, Cd = 0.03 and Pb = 1.23 in year 2.

References

Corbellini M, Canevara MG (1994) Estimate of moisture and protein content in whole grains of bread wheat (*T. aestivum* L.) by near infrared reflectance spectroscopy. Ital J Food Sci 6(1):95-102

Joy EJ, Ander EL, Broadley MR, Young SD, Chilimba AD, Hamilton EM et al. (2017). Elemental composition of Malawian rice. Environ Geochem Hlth, 39(4), 835-845. https://doi.org/10.1007/s10653-016-9854-9

Perten H (1964) Application of the falling number method for evaluating alpha-amylase activity. Cereal Chem, 41(3):127-140.

Supplementary Fig. 1. Histograms of all traits measured in each trial year.

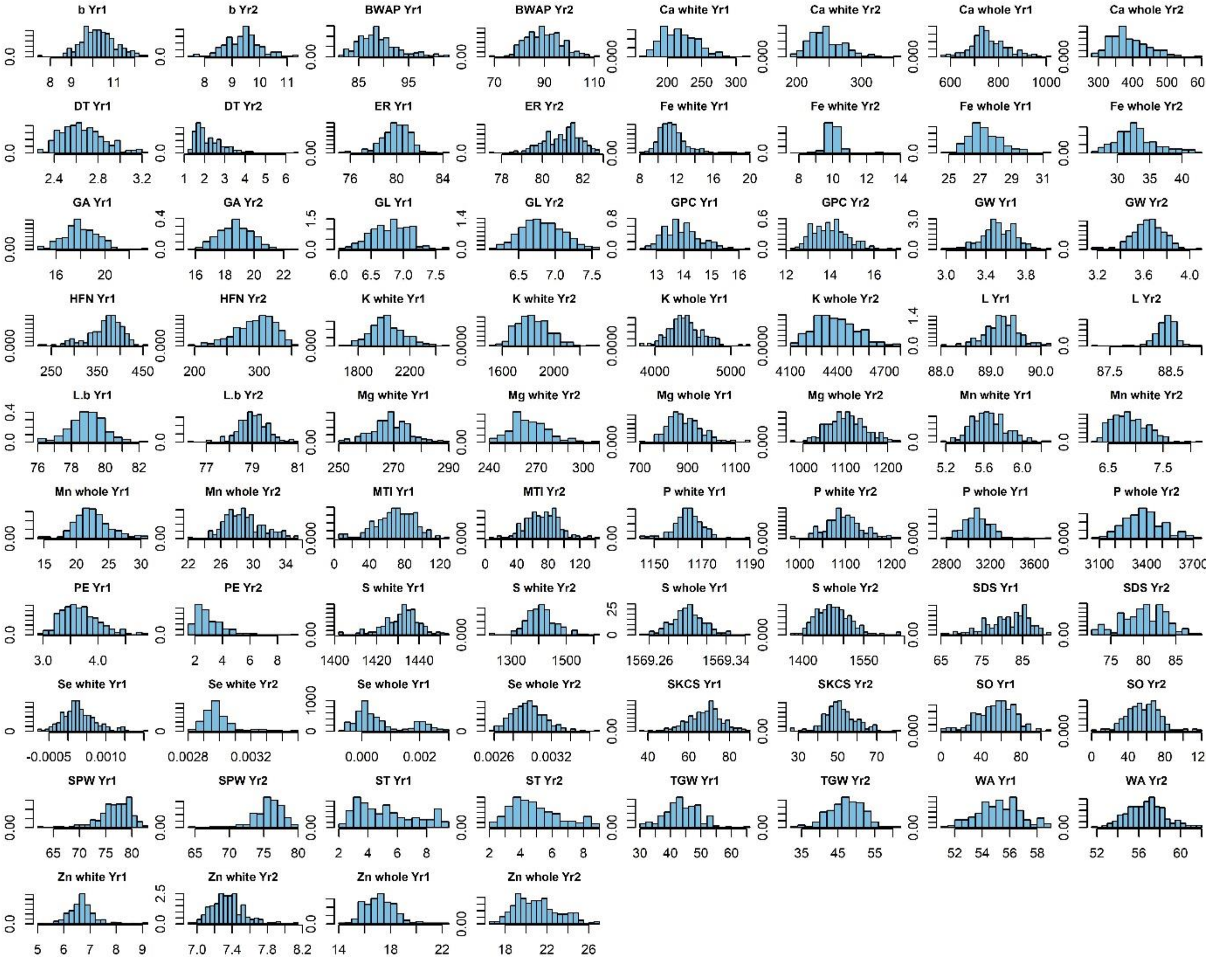

Supplement: Supplementary file 1 — Supplementary Text and Figures [file 41437_2022_503_MOESM1_ESM.pdf]
